# Dealing with Magnetic Disturbances in Human Motion Capture: A Survey of Techniques

**DOI:** 10.3390/mi7030043

**Published:** 2016-03-09

**Authors:** Gabriele Ligorio, Angelo Maria Sabatini

**Affiliations:** The BioRobotics Institute, Scuola Superiore Sant’Anna, Piazza Martiri della Libertà 33, Pisa 56125, Italy; angelo.sabatini@sssup.it

**Keywords:** magnetic sensors, orientation estimation, indoor magnetic disturbances

## Abstract

Magnetic-Inertial Measurement Units (MIMUs) based on microelectromechanical (MEMS) technologies are widespread in contexts such as human motion tracking. Although they present several advantages (lightweight, size, cost), their orientation estimation accuracy might be poor. Indoor magnetic disturbances represent one of the limiting factors for their accuracy, and, therefore, a variety of work was done to characterize and compensate them. In this paper, the main compensation strategies included within Kalman-based orientation estimators are surveyed and classified according to which degrees of freedom are affected by the magnetic data and to the magnetic disturbance rejection methods implemented. By selecting a representative method from each category, four algorithms were obtained and compared in two different magnetic environments: (1) small workspace with an active magnetic source; (2) large workspace without active magnetic sources. A wrist-worn MIMU was used to acquire data from a healthy subject, whereas a stereophotogrammetric system was adopted to obtain ground-truth data. The results suggested that the model-based approaches represent the best compromise between the two testbeds. This is particularly true when the magnetic data are prevented to affect the estimation of the angles with respect to the vertical direction.

## 1. Introduction

Sensing Earth’s magnetic field can aid large-scale navigation. On the one hand, it is known that animals like European robins, turtles or spiny lobsters exploit the Earth’s magnetic field variations across the globe (both in terms of magnitude and inclination) to localize themselves and find the way to their destination [1,2,3]; on the other hand, the compass, which measures the horizontal component of the Earth’s magnetic field, has been the most important direction-finding device since the 11th century [4,5].

Nowadays, sensing Earth’s magnetic field is widely exploited for estimating the three-dimensional (3D) orientation. Aircrafts and ships routinely use expensive Attitude and Heading Reference Systems (AHRS), which include high-grade inertial sensors and compasses [6]. At the same time, however, microelectromechanical systems (MEMS) technologies allow the inclusion of low-grade magnetic sensors in Magnetic-Inertial Measurement Units (MIMUs), as well as in many other mobile devices, including smartphones. Therefore, magnetic MEMS are being successfully employed in several new applications from robotic or unmanned vehicles navigation [7,8] and human motion tracking [9,10,11].

Low-cost, lightweight, small size and ease of use are the main advantages that have contributed to the widespread use of MIMUs [12]. Unfortunately, estimating the 3D orientation from the measurements of the sensors MIMUs contain (a triaxial gyroscope, a triaxial accelerometer and a triaxial magnetic sensor) is a challenging task that often produces low-accuracy estimates, especially during long-lasting experiments [13].

Starting from known initial conditions, the angular velocity measured by a gyroscope can be time-integrated to estimate the 3D body orientation. However, the error sources affecting the gyroscope output (measurement noise, bias, calibration errors, and so forth) lead to drifting integration errors which grow unbounded with time and may also depend on how the MIMU moves in the 3D space [14].

Accelerometers measure the applied specific force, *i.e.*, the sum of the gravity vector and the external acceleration, in the local reference frame. If the external acceleration is assumed to be much smaller than gravity, the angles with respect to the vertical direction (*i.e*., pitch and roll angles, tilt angles or attitude angles) can be computed through standard trigonometrical formulae [15]. Since the accelerometers produce noisy but drift-free angular estimates, they are often coupled to gyroscopes so as to limit the drifting errors along the attitude angles [15,16]. However, the gravity measurement does not convey any information about the angle around the vertical direction, *i.e.* the yaw angle or heading [17].

For this reason, the Earth’s magnetic field, especially its horizontal component, may represent a useful heading reference to aid the gyroscope data integration. However, the Earth’s magnetic field is not constant throughout the globe, both in terms of magnitude and inclination (world magnetic maps can be found in [18]). In particular, the Earth’s magnetic field and the gravity are orthogonal around the equator and parallel in correspondence of the poles. Obviously, when the horizontal component is null, no heading information can be obtained by measuring the Earth’s magnetic field [19]. It is noted that typical contexts where MIMU technology can be applied, such as human motion tracking, do not involve large-scale geographical displacements. Therefore, if the local horizontal component is not null at the locations where the human body has to be monitored, sensing Earth’s magnetic field can be purposefully applied. Nevertheless, the sensed Earth’s magnetic field cannot be considered constant either in this scenario, both in terms of magnitude and inclination. Indeed, ferromagnetic materials and/or magnetic sources produce small-scale magnetic field variations [20], especially in man-made environments. As a consequence, the magnetic field sensed, for example, in a typical interior room setting cannot be taken as a reference [20] to the same extent the gravity is considered for attitude estimation.

In the last years, a lot of work has been performed in order to characterize the magnetic disturbances that are typically present indoors. In [20], it was shown that they are strongly dependent on the distance from the magnetic source. In fact, during experiments involving several types of magnetic sources), it turned out that the magnetic disturbances can exceed the strength of the Earth’s magnetic field few centimeters far from the source, to become negligible at a distance of, say, 1 m. In [21], the spatial distribution of the magnetic disturbance was experimentally mapped within the typical setting of a motion analysis laboratory. The interesting finding was that the homogeneity of the magnetic field was critically dependent on the height from the floor. Specifically, the higher from the floor, the more homogeneous the magnetic field is. This agrees with findings from [22], where this phenomenon was explained by the steel structures that can be present within floors. Moreover, other typical structures like pillars, doors and elevators, add further distortions to the indoor magnetic field [23]. The interesting feature common to all these distorting factors is that the indoor magnetic fingerprint is surprisingly stable, even across months [23].

Therefore, accurately measuring the external magnetic field is not the only requirement for using successfully magnetic sensors. Sophisticated signal processing methods are also needed in order to separate the useful information contained relevant for accurate from the nuisance factors due to the magnetic disturbances.

For this reason, in the last years, the technological advances in the sensing hardware design went together with the development of novel signal processing techniques aimed at interpreting the magnetic sensor output. As proposed in [21], mapping the indoor magnetic field before using the MIMU would help identify and possibly avoid the parts within the workspace that are, magnetically speaking, the most disturbed. However, since this is not always possible, algorithmic strategies are necessary to prevent indoor magnetic disturbances from affecting the performance of orientation estimation algorithms relying on magnetic sensors [20]. From this point of view, this paper is aimed at surveying, analyzing and comparing some promising approaches conceived to mitigate the effect of magnetic disturbances in the determination of the orientation.

In this regard, it is very interesting to note that, in some particular applications like indoor pedestrian localization, the spatial distribution of the indoor magnetic disturbances can be an important information source. In fact, the magnetic field sensed at a given point can be used to estimate the current user location, provided that a magnetic map of the workspace is also available [22,23,24,25]. However, the review of this research topic is out of the scope of this paper, which focuses mainly on the most popular strategies to deal with the magnetic field disturbances when orientation of a rigid body is to be estimated using data from MIMU sensors.

The present manuscript is organized as follows: in Section 2, the rigid body orientation basics are detailed; in Section 3, the state-of-the-art to deal with the magnetic disturbances in the MIMU-based orientation estimation context are surveyed. The experimental setup adopted to test the main magnetic disturbances rejection approaches is described in Section 4, and the respective experimental outcomes are shown in Section 5. Concluding remarks are presented in Section 6. In the Appendix, the two main approaches for magnetic disturbance rejection are detailed.

## 2. Background

### 2.1. Rigid Body Orientation

The orientation of a body (mobile) reference frame {**b**} with respect to a navigation (fixed) reference frame {**n**} can be expressed in several ways. The rotation matrix, the quaternion of rotation and the Euler angles are by in large the most widely used orientation parameterizations. Readers can refer to [26] for a thorough review on this topic.

In this paper, **R***^bn^* and **q***^bn^* are, respectively, the orientation matrix and the quaternion that transform a generic vector **p***^n^* (observed in the navigation frame) in **p***^b^* (observed in the body reference frame). The quaternion convention proposed in [26] was adopted, where the scalar part is put in the fourth quaternion component. Euler angles are used in this paper as well but mainly for visualization or quantification purposes. The “ZYX” convention is adopted (first rotation around the *z*-axis, rotations being considered about local axes), yielding the yaw (*ψ*), pitch (*ϑ*) and roll angles (*ϕ*) [17]:

(1) $$p^{b}=R^{bn}p^{n}=\left[\begin{array}{ccc} 1 & 0 & 0\\ 0 & \cos\varphi & \sin\varphi \\ 0 & \sin\varphi & -\cos\varphi \end{array}\right]\left[\begin{array}{ccc} \cos\vartheta & 0 & -\sin\vartheta \\ 0 & 1 & 0\\ \sin\vartheta & 0 & \cos\vartheta \end{array}\right]\left[\begin{array}{ccc} \cos\psi & \sin\psi & 0\\ -\sin\psi & \cos\psi & 0\\ 0 & 0 & 1 \end{array}\right]p^{n}$$

**Figure 1:**
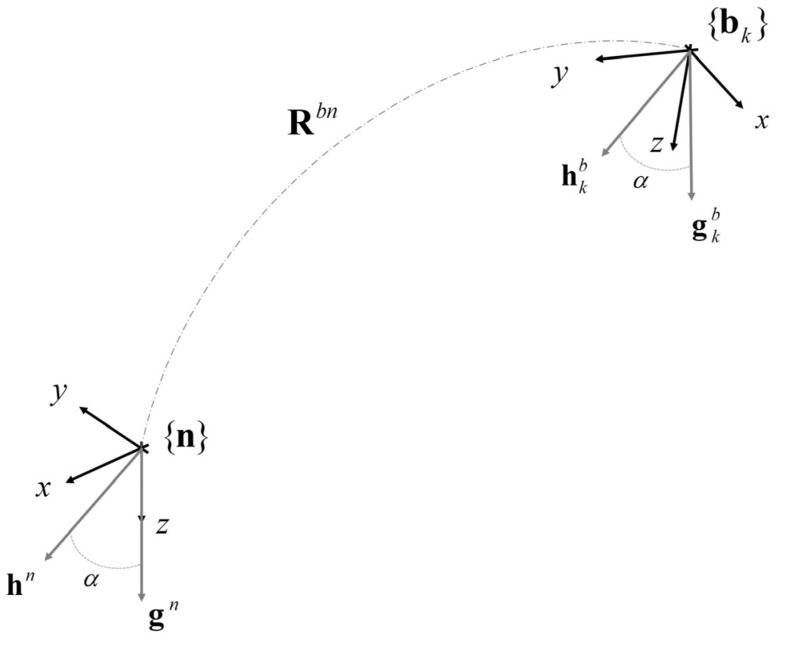
Problem statement: the orientation of {**b**} with respect to {**n**} is described by the rotation matrix **R***^bn^*. The reference vector pairs (**h***^n^*, **g***^n^*) and (**h***^b^*, **g***^b^*) are shown in gray. The angle *α* is defined as the dip angle and it is independent from the reference frame.

The North-East-Down (NED) convention can be adopted to define {**n**} [17]. According to this convention, the *z*-axis is parallel to the vertical direction (pointing downward) and the heading is chosen in order that the Earth’s magnetic field has a null component along the *y*-axis, Figure 1.

As a consequence, the yaw angle represents the rotation about the vertical axis, whilst the pitch and roll angles represent the two tilt angles with respect to the vertical direction. The yaw angle is also known as heading, while the pitch and roll angles are often grouped together to form the attitude angle [9,13].

### 2.2. Orientation Estimation: Single-Frame Approaches

The rotation between two reference frames can be computed if the coordinates of at least two vectors are known in both the reference frames (the so-called Wahba’s problem [27]). The gravity vector and the Earth’s magnetic field are two possible reference vectors that can be exploited for this purpose. In fact, under certain assumptions (see Section 1), the reference vectors can be measured in {**b**} by means of a triaxial accelerometer and a triaxial magnetic sensor. On the other hand, according to the NED convention, their coordinates in {**n**} are:

(2) $$\begin{array}{cc} h^{n}=\left[\begin{array}{c} \sin\alpha \\ 0\\ \cos\alpha \end{array}\right]h, & g^{n}=\left[\begin{array}{c} 0\\ 0\\ 1 \end{array}\right] \end{array}g$$

where *g* is the norm of the gravity vector, *h* is the norm of the Earth’s magnetic field and *α* is the dip angle, *i.e.*, the angle between the two reference vectors which is the complementary angle of the Earth’s magnetic field inclination. While *g* may be assumed constant across the globe, *h* and *α* vary according to the geographic location [18].

The Wahba’s problem solutions are known as single-frame approaches since they do not involve any gyroscope-based frame-by-frame propagation. Many single-frame methods have been proposed [17], among which TRIAD [28] and QUEST [29] are the most popular. For the scope of this paper, their main difference relies in the weight they assign to the measurements of the reference vectors. In particular, in the TRIAD algorithm the reference vector assumed most reliable drives the solution provided to Wahba’s problem. In the case of interest in this paper, the measurement of the gravity is assumed to be more reliable. In practice, this means that the measurement of the Earth’s magnetic field is prevented from affecting the attitude angles. In fact, it is used for the heading estimation only, after the attitude has been already computed. [30]. Conversely, the optimization procedure implemented in the QUEST algorithm allows assigning the same belief to both reference vectors [10]. Hence, all sensory information is exploited during vector matching. In practice, this means that magnetic disturbances can affect the estimation of either attitude or heading angles.

### 2.3. Kalman Filtering: Basics

As mentioned in Section 1, gyroscope, accelerometer and magnetic sensor data are often used together to estimate the 3D rigid body orientation because of their complementary properties [31]. In this regard, Kalman filtering is the most popular approach for pursuing sensor fusion.

Suppose that the following generic time-variant linear system model is built:

(3) $$\begin{array}{l} x_{k}=F_{k-1}x_{k-1}+G_{k}u_{k}\\ y_{k}=H_{k}x_{k} \end{array}$$

The aim of Kalman filtering is to optimally estimate (in a least-square sense) the state vector **x*_k_*** given the knowledge of the system model (**F***_k−1_*, **G***_k_* and **H***_k_*), the input vector **u***_k_* and the measurement vector **y***_k_* [32].

The estimation takes place in two recursive steps [32]. In the prediction step (or time update), the state vector is predicted according to the previous state vector estimate, the system prediction model and the input vector. In the measurement update step, the predicted state vector (*a priori* estimate) is corrected by means of the last measurement vector available. In the Kalman framework, the predicted state vector and the measurement vector are weighted with the Kalman gain, which depends both on the system model and on data uncertainties [32], to produce the corrected state vector (*a posteriori* estimate). Since multiple measurements can be inserted either in the input vector or in the measurement vector, Kalman filtering represents a framework that is very well suited to fuse disparate sensor data.

A considerable freedom exists when designing a Kalman Filter (KF). In fact, different system models, *i.e.*, different definitions of the state, input and measurement vectors, lead to different Kalman equations. In addition, it is noted that the system model could be even nonlinear. To cope with the nonlinearity, several strategies are available, *i.e.*, Extended Kalman Filters (EKF), Unscented Kalman Filters (UKF), Indirect Kalman Filter (IKF), yielding even further flexibility in the design of the sensor fusion algorithm [32].

When Kalman filtering is adopted for the orientation estimation based on MIMU sensor data, the standard approach is to consider the quaternion of rotation being part of the state vector [33,34,35]. Other ancillary quantities, such as inertial sensor biases or the angular velocity itself, can also be included in the state vector [10,19]. Gyroscope measurements may be considered either as an input [17,35] or as a measurement [10]. On the other hand, accelerometer and magnetic sensor data are always considered as measurements [15,17,35].

## 3. Orientation Estimation: How to Deal with the Problem of Magnetic Disturbances

**Figure 2:**
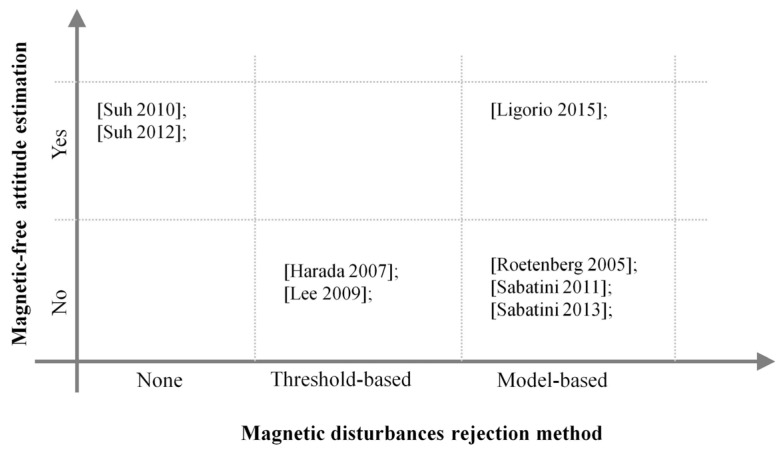
The orientation estimator classification scheme proposed in this paper. The most popular approaches to deal with the magnetic disturbances are placed within this scheme.

In this section, the main Kalman-based sensor fusion approaches developed to deal with the problem of magnetic disturbances in the context of the 3D orientation estimation are reviewed. In particular, two algorithm classifications are considered: the first one considers solutions where magnetic sensor data are either considered or not for estimating the attitude; the second one regards the methods of magnetic disturbance rejection implemented, which can be either threshold-based or model-based. In Figure 2, the classification scheme adopted in this paper is shown.

### 3.1. Magnetic-Free Attitude Estimation

As explained in Section 1, the main reason for embedding magnetic sensors within MIMUs is related to the opportunities for accurate heading estimation. However, magnetic sensor data convey information concerning attitude as well, due to the Earth’s magnetic field vertical component. Depending on the algorithm design, this information may be employed or not for estimating the attitude. Magnetic sensor data are sometimes prevented from computing the attitude for two main reasons: (1) the measurement of the Earth’s magnetic field is usually believed less reliable than the measurement of the gravity acceleration [20]; (2) in principle, the estimation of attitude can be performed using inertial sensors only. As mentioned above (Section 2.2), the TRIAD method adopts this approach.

Regarding the Kalman-based methods, in [36] an IKF was presented where the attitude information carried in magnetic sensor data was discarded using a two-step measurement update. In the first step the accelerometer measurement update was performed in a standard way, leading to attitude estimation. Then, the magnetic measurement update took place, leading to heading estimation. In [37], a simpler approach was proposed by the same authors. It consisted of a standard one-step measurement update where the magnetic sensor data were pre-processed using the accelerometer data, so as to cancel their influence on the attitude estimation process.

### 3.2. Threshold-Based Approaches for Magnetic Disturbance Rejection

Exploiting some *a priori* knowledge about the Earth’s magnetic field is one of the possible strategies to reject disturbed magnetic measurements. In particular, some features of the sensed magnetic field can be computed and compared with those that are expected based on the available *a priori* knowledge of the Earth’s magnetic field. If large differences are detected (exceeding carefully chosen threshold values), the actual measurement is discarded. The strength and the dip angle of the sensed magnetic field are the most used features in this regard.

This solution is easy to implement and do not require relevant computational efforts. However, the threshold tuning process is usually troublesome and the behavior of the algorithm can be somewhat erratic for values of the magnetic features close to the threshold values.

In [38], a UKF was presented where accelerometer and magnetic sensor measurements were used to correct the gyroscope-based quaternion prediction. However, before performing the update step, the magnetic sensor output was checked. The difference between the norm of the sensed magnetic field and the reference magnetic field was compared to a threshold, as well as the estimated dip angle. If one of these differences exceeded the respective threshold, the magnetic sensor data was assumed unreliable and then discarded. In addition, even if the actual measurement was accepted, the measurement noise covariance matrix was adapted to the difference. As a consequence, the confidence in the samples that differed more from the filter prediction was decreased. In [35], a linear KF was implemented, where the quaternion predicted by the gyroscope data was updated with the quaternion provided by the QUEST algorithm. However, the magnetic sensor data (as well as the accelerometer data) had to be compared with a threshold as in [38]. If the current measurement was discarded, the predicted magnetic field (or the predicted gravity) was used as an input for the QUEST algorithm instead of the sensed magnetic field.

### 3.3. Model-Based Approaches for Magnetic Disturbance Rejection

As opposed to the threshold-based approaches, the model-based approaches for magnetic disturbances rejection take explicitly into account that the magnetic sensor data are systematically corrupted by undesired components, which add to the Earth’s magnetic field. Therefore, the attempt is made to model and estimate the magnetic disturbances at each iteration step of the filtering algorithm in order to compensate the raw measurements. In this way, data did not have to be compared to any threshold, although more computational power is required because the state vectors have to be augmented with extra components.

One of the first model-based rejection methods was presented in [39], where three extra state components of the proposed IKF were devoted to estimate the magnetic disturbances, assuming a first-order Gauss-Markov (GM) dynamics. In this way, the magnetic sensor output was compensated (assuming an additive model) before being used to update the predicted state vector. A simple experiment was then performed to show that the compensated IKF was robust against the applied magnetic disturbances. In fact, the same estimation accuracy was obtained in both a disturbed and an undisturbed magnetic environment. The same dynamical disturbance model was adopted in the EKF proposed in [17]. However, besides the model-based compensation, the magnetic data reliability was assessed by comparison with the magnetic field predicted by the EKF. If the norm of the difference exceeded a certain threshold, the magnetic sample was discarded. In [40], a variable-state dimension EKF was proposed instead. Under low (or null) magnetic disturbances, the first-order GM model was employed. However, when high disturbances were detected, three additional state components were added to the state vector in order to implement a second order GM model, until the low magnetic disturbance condition was restored. The magnetic disturbance detection was based on the difference between the measured and expected magnetic field, as in [17].

In all of the Kalman-based methods reviewed above, the state vectors included some components for the rotation estimation (typically quaternions of rotation or orientation errors) and some components for the magnetic disturbance estimation. Conversely, in [9], a novel approach was proposed to deal with the orientation estimation under magnetic disturbances. A linear KF was adopted to separate the Earth’s magnetic field and the magnetic disturbances from the magnetic sensor outputs. These two contributions were directly included within the state vector, and predicted by means of the gyroscope data and a first-order GM model, respectively. The linear and time-invariant measurement model explained the magnetic sensor data as the sum of the two magnetic vectors. At the same time, another identical KF performed the separation between the gravity and the external acceleration on the accelerometer data. Therefore, the two reference vectors, *i.e.*, the gravity and the Earth’s magnetic field, were used to feed the cascaded TRIAD algorithm, so as to perform the final orientation estimation. The novelty of such an approach is that the actual orientation estimation (which is a nonlinear process) is postponed to a second step, after that the Kalman filtering takes place in a linear framework.

## 4. An Experimental Proof of Concept

In this section, the different approaches to deal with the magnetic disturbances in the context of the orientation estimation reviewed above are tested on real human motion data. In particular, the algorithms presented in [9,35] are considered as representatives of the threshold-based and model-based approaches, respectively. Both the algorithms rely on linear Kalman filtering and include a single-frame method for the quaternion computation. For readers’ convenience, these two algorithms are reviewed in the following sections.

### 4.1. Considered Orientation Estimation Methods

In this paper, the orientation estimators presented in [9,35] were taken as representatives of the threshold-based and model-based magnetic disturbance approaches. For the sake of readers’ convenience they are reviewed in detail in the Appendix. These algorithms both rely on linear Kalman filtering. In fact, the nonlinearity of the relationship between the reference vectors and the quaternion of orientation is treated within the respective single-frame method, which may be either before or after the Kalman filtering step. The main differences between the two approaches are: (1) in [35], the body acceleration and the magnetic disturbances are rejected with a threshold-based approach, whereas in [9] a model-based approach is used instead; (2) in [35], the QUEST algorithm is adopted, allowing magnetic measurement to affect the whole output, whereas in [9], the magnetic measurement is excluded from the attitude computation due to the TRIAD algorithm.

**Figure 3:**
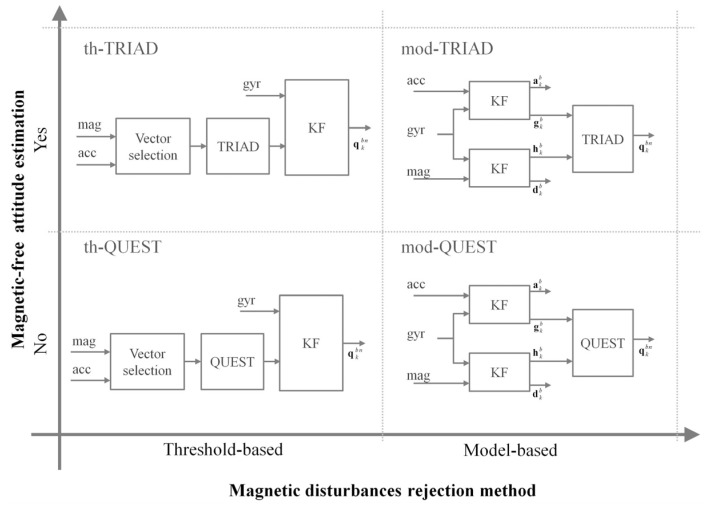
Methods considered in this work for an experimental comparison. The algorithms presented in [9,35] were taken as representatives of the threshold-based and the model-based approaches, respectively. Then, they were combined with the TRIAD and QUEST single-frame methods in order to span all the possible combinations.

In order to evaluate the contribution of these factors separately, two additional methods were considered in the comparisons performed in this work. They were obtained by switching the single-frame methods in both algorithms, spanning all the four combinations shown in Figure 3. Hereafter, the algorithms are named according to the magnetic disturbances rejection approach they implement (th- or mod-) and the single-frame method (TRIAD or QUEST), Figure 3.

As for all the threshold-based approaches, in [35], some gating parameters had to be set. Tuning such parameters consisted in trading-off the number of samples accepted in the algorithm and their quality. Accepting a low number of magnetometer and/or accelerometer readings might yield high-quality but insufficient information, yielding drifting errors. On the other hand, inaccurate estimation might result from processing too much corrupted samples. Usually the tuning process consists in trial and error procedures, where the threshold values were fine tuned according to the output errors.

Conversely, the tuning parameters required by the method proposed in [9] were *c_a_* and *c_b_*. In [15], a grid search was performed in order to obtain the values which minimized the errors with respect to a ground-truth motion. In this work, the same values proposed in [15] granted a good performance in all the cases considered (*c_a_* = 0.1; *c_b_* = 1).

### 4.2. Experimental Setup

In the experiment performed in this paper, one adult healthy subject wore an Opal MIMU (Opal, APDM Inc., Portland, OR, USA) containing 3D gyroscopes, accelerometers and magnetic sensors (gyroscope: full scale ± 1500°/s, sensitivity 0.5 mV/(°·s^−1^), noise density 20 μV(°·s^−1^)/$\sqrt{\mathrm{Hz}}$; accelerometer: full scale ± 6 g, sensitivity 0.22 V/g, noise density 50 μg/$\sqrt{\mathrm{Hz}}$ with g = 9.81 m/s^2^; magnetometer: full scale ± 600 μT, sensitivity 1mV/Gauss, noise density 50 nV/$\sqrt{\mathrm{Hz}}$) on the right (dominant) wrist. The unit was equipped with four reflective InfraRed (IR) markers to acquire ground-truth data from a stereophotogrammetric motion capture system. The optical body reference frame (defined by the markers) and the unit reference frame were preliminarily aligned with the procedure described in [41].

The standard deviations of the sensors measurement noise were evaluated from a data series acquired while the unit was kept still for a minute. In fact, for the algorithms being considered, the information about the sensor measurement noises is required both by the process and/or measurement models and the Kalman calculations. The values obtained were 0.5°/s, 0.02 m/s^2^ and 0.15 µT for the gyroscope, the accelerometer and the magnetometer, respectively.

Two experimental scenarios were considered as testbeds for the considered algorithms. In the first one, the subject was asked to perform a set of manual routines—drinking a glass of water (5 s), writing with a pencil (5 s), writing using a keyboard (5 s), brushing teeth (10 s), brushing hair (10 s), reaching towards a small magnet placed on the table (5 s), and moving an object from the table to the lower shelf and back to the table (8 s)—interlaced with 2 s pauses, while seated. In the second one, the subject walked for three minutes along an eight-shaped path in order to stay within the cameras field of view.

The two scenarios, *i.e*., the manual routines task and the gait task, had two opposite characteristics from the magnetic environment standpoint. In the former, the workspace was small (0.8 m × 0.8 m), making the assumption of the constant magnetic field more reliable. However, the small magnet produced a high disturbance when the sensor was approached. In the latter, although no active magnetic sources were placed, variations of the sensed magnetic field were expected due to the large workspace explored (4 m × 2 m).

Each sensor was calibrated as described in [13] before performing the experiments. A small static pause was performed at the beginning of the experiment in order to initialize the reference fields and to capture the static bias of the gyroscopes.

The orientation estimates obtained by the four considered methods were compared with the ground-truth orientation in terms of heading and attitude. In particular, the Root Mean Square Errors (RMSEs) were computed as a function of time in order to identify the possible effect of the magnetic disturbances on the orientation estimation:

(4) RMSE(k)head=1k∑i=​1k(2cos[S(Δheadqibn)])2RMSE(k)att=1k∑i=​1k(2cos[S(Δattqibn)])2

where the Δheadqibn and Δattqibn encoded the orientation error related to the heading and the attitude of of $\Delta q_{i}^{bn}$ (the error quaternion which aligned the IR marker frame to the MIMU reference frame) and the *S*(**q**) operator extracts the scalar part from the quaternion **q**.

## 5. Results and Discussions

### 5.1. Orientation Estimation Results: Manual Routines Task

**Figure 4:**
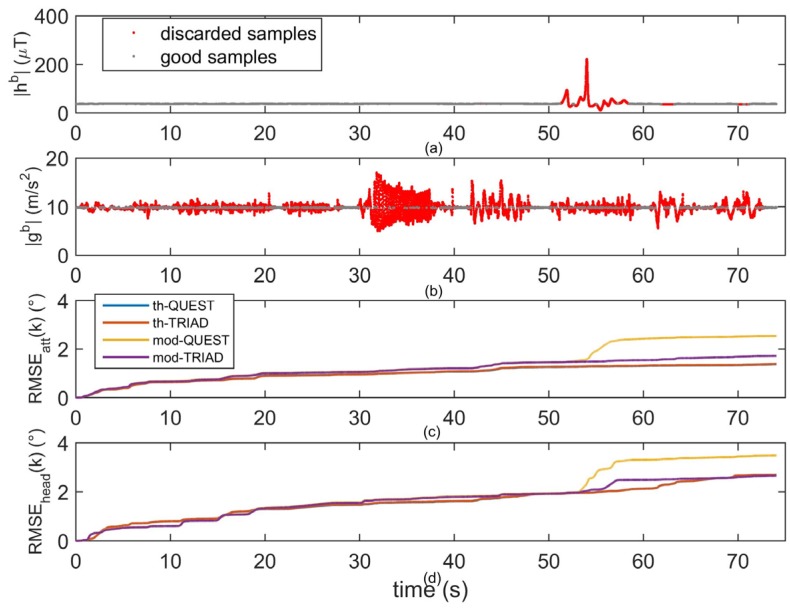
Comparison of the considered methods during the manual routines task: (**a**) norm of the sensed magnetic field; A magnetic disturbance is clearly visible when the sensor was approached to the magnet; (**b**) Norm of the acceleration measurements; (**c**) RMSE along the heading as a function of time for the four algorithms; (**d**) RMSE along the attitude as a function of time for the four algorithms. In (**a**) and (**b**), the samples accepted by the threshold-based algorithms are reported in gray, while the discarded samples are reported in red.

In Figure 4, the results obtained during the manual routines task were shown. Due to the small workspace, the norm of the magnetic field was constant during the task execution. However, from 50 to 60 s, the unit was approached to the small magnet and a very large disturbance was detected. In Figure 4a, the red samples represented the highly disturbed magnetic samples which were discarded by the threshold-based methods. Similarly, in Figure 4b, the red samples represented the accelerometer measurements discarded by the vector selection block because they contained a too large contribution due to the body acceleration. Before the magnetic disturbance (from 0 to 50 s) the four algorithms showed a very similar performance in the attitude and heading estimation, Figure 4c,d. On the contrary, in correspondence of the magnetic disturbance (from 50 to 60 s) the RMSE*_att_* and RMSE*_head_* relative to the model-based methods increased significantly (about 1° or more) with respect to the other methods. Hence, very large disturbances as the one detected in this trial (the magnetic disturbance was much larger if compared to the Earth’s magnetic field norm, Figure 4a) could not be accurately rejected by the model-based methods relying on a simple first-order model as the one implemented in [9]. Switching to higher order models could be a possible solution to this issue [40]. However, as opposed to mod-QUEST, the attitude estimates provided by mod-TRIAD was completely insensitive to the poor estimates of the disturbances due to the way the TRIAD algorithm works (see Section 2.2), which excludes the magnetic measurements from the attitude estimation. In fact, the performance of the mod-TRIAD for the attitude was almost identical to the one of the threshold-based methods. However, this is not true for the heading output of mod-TRIAD, which was affected by the bad magnetic measurements, although not as severely as the mod-QUEST algorithm.

By looking at Figure 4c,d, it can be concluded that using the magnetic field for the attitude estimation when no disturbances were detected did not produce any advantage for the attitude. This might be due to the fact that accelerometer measurements were relatively reliable, mainly because the static pauses between the sub-tasks considered in the manual routines. During such pauses, the threshold-based approaches could accept several samples to effectively bind the gyrosocope integration drift. In addition, the model-based approaches took advantage of the static pauses, given that they showed the same attitude accuracy of the threshold-based methods before the disturbance took place. Therefore, it can be concluded that the magnetic measurements should be excluded from the attitude estimation whenever the accelerometer reading can be regarded as a reliable estimate of the gravity. In fact, no advantage was observed when the magnetic environment was good. On the contrary, the corrupted magnetic measurements produced a detrimental effect when the magnetic environment was disturbed.

It should be mentioned that the manual routines task was somehow best-suited for the threshold-based approaches because the magnetic disturbance was detected only in a short time window. In these situations, discarding the magnetometer data is a simple and effective solution. However, if the magnetic disturbance was detected for longer time windows, there would be no suitable information to aid the heading estimates longer periods, leading to possible drifting errors.

### 5.2. Orientation Estimation Results: Gait Task

**Figure 5:**
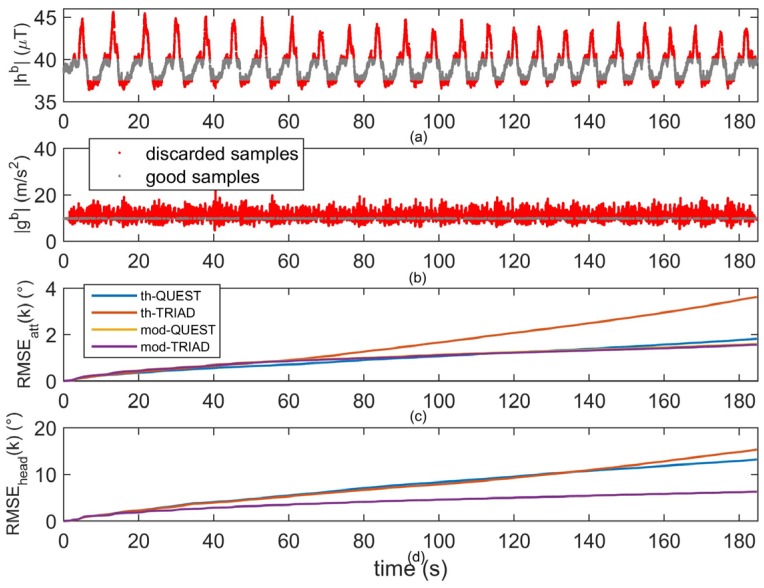
Comparison of the considered methods during the gait task: (**a**) norm of the sensed magnetic field. A magnetic disturbance pattern, related to user’s displacements within the workspace; (**b**) norm of the acceleration measurements; (**c**) RMSE along the heading as a function of time for the four algorithms; (**d**) RMSE along the attitude as a function of time for the four algorithms. In (**a**) and (**b**), the samples accepted by the threshold-based algorithms are reported in gray, while the discarded samples are reported in red.

During the gait task, a relatively large workspace was explored, see Section 4.2. For this reason, as depicted in Figure 5a, the norm of the sensed magnetic field was not constant, showing a quasi-periodic trend instead. Such effect is due to the periodicity of the eight-shaped path the subject was asked to go through. The clear relationship between the norm of the sensed magnetic field and the subject’s spatial position suggested that, in the experiment performed in this work, small-scale variations of the indoor magnetic field were involved, similarly to the ones found in [21,22,24].

The threshold-based approaches discarded several samples (black samples in Figure 5a) because they were too different from the reference value, taken at the subject’s initial position. In addition, most of the accelerometer samples were discarded as well because the motion was relatively fast for all the trial and no static pauses were involved, as opposed to the manual routines tasks. The performance of the four algorithms when estimating the attitude was quite similar, except for the th-TRIAD method which final error was about 1.5° larger than the other methods, Figure 5c. Apparently, the th-QUEST method took advantage from the magnetic samples (only the ones accepted by the vector selection step) for the attitude estimation. As opposed to the manual routines task, the accelerometer measurements did not provide a reliable reference for the attitude estimation. Therefore, even if partially corrupted and discontinuous, the magnetic samples proved to be useful to the threshold-based methods to improve the attitude estimation. On the contrary, the attitude information in the magnetic field did not produce any advantage in the model-based approaches. In fact, the mod-TRIAD and mod-QUEST algorithms obtained the same accuracy. This result suggested that the model-based processing performed on the accelerometer samples was more effective than the simple thresholding.

For what concerns the heading estimation (Figure 5d), the model-based approaches performed considerably better than the threshold-based approaches. A possible explanation is that the threshold-based approaches discarded several magnetic samples. For this reason, there might be not enough information to bind the gyroscope integration drift. Increasing the threshold values would allow for including more magnetic data in the KF. However, more distorted samples would be considered, leading to inaccurate estimates. On the other hand, all the samples were processed in the model-based approaches. As in the accelerometer case described above, the continuous model-based compensation allowed one to reliably use the magnetometer to correct the gyroscope predictions. Finally, the slight improvement of th-QUEST over the th-TRIAD for the heading might be due to the better attitude estimation, in accordance with [42].

## 6. Conclusions

In this paper, the problem of estimating the 3D rigid body orientation with MIMU data was treated with a particular attention due to the indoor magnetic field disturbances issue. In particular, the main magnetic disturbances rejection approaches (*i.e*., threshold-based and model-based) were reviewed and four representative algorithms were selected and compared. From the experimental comparison, it turned out that the threshold-based approach might be preferable when very high disturbances are experienced for a short amount of time. On the other hand, when low disturbances are encountered throughout the trial, the model-based methods outperform the threshold-based ones, which discard too many samples. Another issue with the threshold-based approaches is that the results may be strongly affected by the selected thresholds. On the whole, the model-based approach represents the best compromise, especially when the magnetic measurements are prevented from affecting the attitude estimation. In fact, this solution produced very good results in both the considered experimental scenarios.

## Figures and Tables

**Figure A1:**
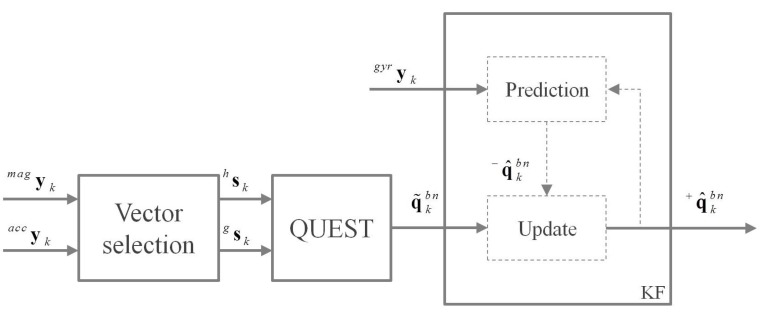
Overall workflow of the orientation estimator proposed in [35].

**Figure A2:**
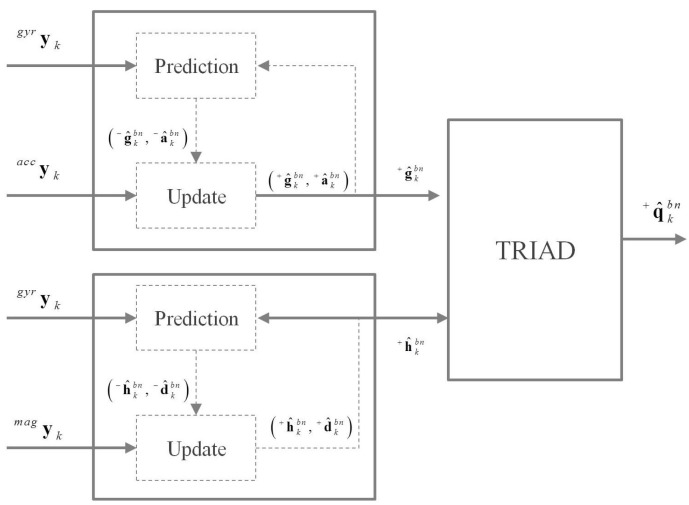
Overall workflow of the orientation estimator proposed in [9].

## Appendix

In this appendix, the orientation estimators taken as representatives of the threshold-based and the model-based magnetic disturbance rejection methods were detailed.

Hereafter, $\hat{p}$ and $\tilde{p}$ denote an estimate and a measurement of **p**, respectively. In the Kalman filtering context p^− and p^+ represent the *a priori* and the *a posteriori* estimates of **p**. Finally, **I_n_** and **0**_n_ stand for the n × n identity and null matrices, respectively.

### A.1. Selected Threshold-Based Method

In [35], the quaternion of orientation $q_{k}^{bn}={\left[\begin{array}{cccc} q_{1,k} & q_{2,k} & q_{3,k} & q_{4,k} \end{array}\right]}^{T}={\left[\begin{array}{cc} {\bar{q}}_{k} & q_{4,k} \end{array}\right]}^{T}$ represented the state vector, as shown in the dynamic model reported in Equation (A1). In addition, it was assumed that it was directly measured from the system output (Equation (A2)):

(A1) $$q_{k}^{bn}=\Phi_{k-1}q_{k-1}^{bn}+w_{k-1}$$

(A2) $${\tilde{q}}_{k}^{bn}=q_{k}^{bn}+v_{k}$$

with

(A3) $$\Phi_{k-1}=I_{4}+T_{s}\left[\begin{array}{cc} -\left[\omega_{k-1}\times \right] & \omega_{k-1}\\ -\omega_{k-1}^{T} & 0 \end{array}\right]$$

(A4) wk−1=−Ts2Ξk−1nk−1gyr

where $\omega_{k-1}={\left[\begin{array}{ccc} \omega_{x,k-1} & \omega_{y,k-1} & \omega_{z,k-1} \end{array}\right]}^{T}$ is the angular velocity measured by the gyroscope at the *k*-th time instant, *T_s_* is the sampling time, nk−1gyr is the measurement noise of the gyroscope, **v*_k_*** is the measurement noise and

(A5) $$\left[\omega_{k-1}\times \right]=\left[\begin{array}{ccc} 0 & -\omega_{z,k-1} & \omega_{y,k-1}\\ \omega_{z,k-1} & 0 & -\omega_{x,k-1}\\ -\omega_{y,k-1} & \omega_{x,k-1} & 0 \end{array}\right]$$

(A6) $$\Xi_{k-1}=-\frac{T_{s}}{2}\left[\begin{array}{c} \left[{\bar{q}}_{k-1}\times \right]+q_{4,k-1}I_{3}\\ -{\bar{q}}_{k-1}^{T} \end{array}\right]$$

At each iteration the *a priori* quaternion q^kbn− was computed according to the gyroscope measurements and the prediction Equation (A1). Then, an additional measurement of the current quaternion of orientation ${\tilde{q}}_{k}^{bn}$ was obtained from the QUEST algorithm, fed with accelerometer and magnetometer data, Figure A1. The predicted and the measured quaternions were then fused together according to the standard Kalman equations to produce the output quaternion.

In Figure A1, it was shown that the accelerometer and magnetometer data were assessed in the vector selection block before being used into the QUEST algorithm. In this block, the norm of the current accelerometer and magnetometer readings, as well as the dip angle, were checked against their expected and/or initial values [35]. Possible corrupted samples were then substituted with their *a priori* estimates:

(A7) sg={ykacc‖ykacc‖ , if|‖ykacc‖−‖gn‖|<thaccq^kbn−⊗[gn0]T⊗q^knb−, otherwise

(A8) sh={ykmag‖ykmag‖ , if|‖ykmag‖−‖m0‖|<thacc∩|αk−α0|<thαq^kbn−⊗[hn0]T⊗q^knb−, otherwise

where ⊗ and ‖ ‖ represent, respectively, the quaternion multiplication and the norm operators, **s**_g_ and **s***_h_* are, respectively, the gravity and magnetic field input signals of the QUEST algorithm (see Figure A1), *^acc^***y***_k_* and *^mag^***y***_k_* are, respectively, the *k*-th accelerometer and magnetometer sample, **th***_acc_*, **th***_mag_* and **th***_α_* are, respectively the tsshreshold values adopted for the accelerometer samples norm, the magnetometer samples norm and the dip angle.

As shown in Equations (A7) and (A8) three threshold parameters needed to be set in [16], *i.e*., **th***_acc_*, **th***_mag_* and **th***_α_*. A trade-off value was found between drifting behaviors (arising from being too strict in the acceptance of accelerometer and magnetometer data) and distortions (due to unfiltered body acceleration and/or magnetic disturbances). The chosen values were reported in Table A1.

**Table A1:** Acceleration, magnetic field and dip angle values adopted in the vector selection performed in the threshold-based approaches.

th_*acc*_	th_*mag*_	th_*α*_
0.08 m/s^2^	1.20 µT	5°

### A.2. Selected Model-Based Method

In [15], a KF was presented which aimed at the gyroscope-aided separation of the gravity and body acceleration contributions ($g_{k}^{b}$ and $a_{k}^{b}$, respectively) from the accelerometer signal. The state vector was composed of these two vectors, and their time evolution was modeled with the following dynamic linear system:

(A9) xk=[gkbakb]=[exp([ωk−1×]Ts)0303I3⋅ca][gk−1bak−1b]+[−[gk−1b×]0303I3⋅cb][nk−1gyrnk−1GM]

(A10) $$y_{k}=\left[\begin{array}{cc} I_{3} & I_{3} \end{array}\right]\left[\begin{array}{c} g_{k}^{b}\\ a_{k}^{b} \end{array}\right]+v_{k}$$

where exp(·) is the exponential matrix operator, *C_a_* and *C_b_* are the two parameters of the Gauss-Markov (GM) model adopted for the propagation of the body acceleration and nk−1GM is white standard noise.

The gravity and the body acceleration were predicted according to the gyroscope measurements and the first-order GM model Equation (A9). Then, they were merged with the information from the measurement model Equation (A10) and the accelerometer samples to provide the *a posteriori* estimates g^kb+ and a^kb+.

As shown in Figure A2, in [9] this idea was further extended to the magnetometer measurements as well, in order to separate the Earth’s magnetic field and the magnetic disturbance contributions ($h_{k}^{b}$ and $d_{k}^{b}$, respectively) from the magnetometer readings. The two KFs were run in a parallel way in order to use the two *a posteriori* estimates g^kb+ and h^kb+ to feed a vector matching algorithm (provided that **g*^n^*** and **h***^n^* are known) and compute the body orientation. In particular, in [9], the TRIAD algorithm was chosen because it prevented the magnetic measurements affecting the attitude estimation.
